# Microsatellite Instable Colorectal Adenocarcinoma Diagnostics: The Advent of Liquid Biopsy Approaches

**DOI:** 10.3389/fonc.2022.930108

**Published:** 2022-06-28

**Authors:** Carlotta Ceccon, Valentina Angerilli, Cosimo Rasola, Letizia Procaccio, Marianna Sabbadin, Francesca Bergamo, Umberto Malapelle, Sara Lonardi, Matteo Fassan

**Affiliations:** ^1^ Department of Medicine (DIMED), University of Padua, Padua, Italy; ^2^ Veneto Institute of Oncology, IOV-IRCCS, Padua, Italy; ^3^ Department of Surgery, Oncology and Gastroenterology, University of Padua, Padua, Italy; ^4^ Department of Public Health, University of Naples Federico II, Naples, Italy

**Keywords:** liquid biopsy, colorectal cancer, microsatellite Instability, immunotherapy, circulating tumor DNA, cancer screening, early detection

## Abstract

The introduction of immunotherapy has revolutionized the oncological targeted therapy paradigm. Microsatellite instability (MSI) identifies a subgroup of colorectal cancers (CRCs) which respond to treatment with immune checkpoint inhibitors. Tissue biopsy is currently the gold standard for the assessment of MSI/Mismatch Repair deficiency (MMRd) by means immunohistochemistry or molecular assays. However, the application of liquid biopsy in the clinic may help to overcome several limitations of tissue analysis and may provide great benefit to the diagnostic scenario and therapeutic decision-making process. In the context of MSI/MMRd CRC, the use of liquid biopsy may allow to establish MSI/MMR status if tissue sampling cannot be performed or in case of discordant tissue biopsies. Liquid biopsy may also become a powerful tool to monitor treatment response and the onset resistance to immunotherapy over time and to stratify of MSI/MMRd patients according to their risk of relapse and metastases. The aim of this review is to summarize the main technical aspects and clinical applications, the benefits, and limitations of the use of liquid biopsy in MSI/MMRd colorectal cancer patients.

## Background

With 1.9 million new cases and almost 0.9 million deaths in 2020, colorectal cancer (CRC) accounts for approximately 10% of all cancers and cancer-related deaths (1).

In recent years, remarkable progress has been made in the development of biomarker-driven targeted therapies, revolutionizing the treatment scenario for patients with metastatic CRC (mCRC).

CRCs can be categorized into two discrete subgroups: those with a microsatellite instability (MSI)/Mismatch Repair Deficiency (MMRd) signature (~15%) and those with a microsatellite stability (MSS)/Mismatch Repair Proficiency (MMRp) signature (~85%) (2).

MSI is the molecular fingerprint of MMRd. Microsatellites are repetitive sequences distributed throughout the human genome, which are especially prone to the accumulation of mutations (3).

MMR is a highly conserved protein complex that plays a key role in maintaining genomic stability, by correcting short deletions and single base mismatches that can develop during DNA replication and recombination. The most important MMR proteins include MLH1 (mutL homologue 1), MSH2 (mutS homologue 2), MSH6 (mutS homologue 6) and PMS2 (post-meiotic segregation increased 2) and function in heterodimers (MLH1 and PMS2, MSH2 and MSH6) (4).

The loss of function of the MMR genes can be caused by germline and/or somatic mutations or epigenetic silencing (usually *MLH1* gene promoter hypermethylation), resulting in increased mutational burden (5). Approximately 2-3% of CRCs are caused by a germline mutation of MMR proteins, which is the genetic hallmark of Lynch Syndrome (6).

The MSI/MMRd tumor phenotype is characterized by a significant intra- and peri-tumoral lymphocytic infiltrate and morphologic heterogeneity (7). MSI/MMRd colorectal adenocarcinoma are often mucinous and rare histotypes such as medullary carcinoma and signet-ring cell adenocarcinoma are not infrequent (8).

The presence of MSI/MMRd has a favorable prognostic value. Locally advanced MSI/MMRd CRCs have a lower risk of recurrence, with a hazard ratio (HR) estimate for overall survival (OS) associated with MSI of 0.65 (95% confidence interval [CI], 0.59 to 0.71) in pooled analysis (9). For this reason, MSI/MMRd tumors represent only ~2–4% of all mCRCs; however, MSI/MMRd is associated with a dismal prognosis in the metastatic setting (progression free survival [PFS]: HR, 133 and 95% CI, 1.12-1.57; OS: HR, 1.35; 95% CI, 1.13-1.61) (10).

MSI/MMRd has also a well-established predictive value. Following some contradictory results, MSI/MMRd has emerged as a predictor of 5-fluorouracil adjuvant therapy among stage II/III colorectal cancer (11, 12). The revolution brought by immunotherapy to the therapeutic landscape of mCRC began with a landmark clinical trial that demonstrated the benefit of anti-PD-1 antibody pembrolizumab in MSI/MMRd tumors (13). In 2017, on the basis of the compelling data of phase II clinical trial CheckMate 142, the Food and Drug Administration (FDA) approved second-line pembrolizumab and nivolumab for patients with MSI/MMRd mCRC (14). In 2020, after phase III clinical trial Keynote-177 demonstrated that pembrolizumab led to significantly longer PFS than chemotherapy as first-line therapy for MSI/MMRd mCRC (HR, 0.60; 95%CI, 0.45 to 0.80) (15), pembrolizumab was approved for previously untreated MSI/MMRd advanced unresectable or mCRCs.

The assessment of MMR status by immunohistochemistry (IHC) is recommended in all CRC patients and in all patients with any cancer type belonging to the spectrum of cancers found in Lynch syndrome. In clinical practice, IHC is preferred over PCR-based MSI testing, because of its lower turnaround time and lower costs and high concordance rate between the two methods (16). MMR IHC interpretation is usually straightforward, but challenges can be occasionally encountered, including tumor staining weaker than control, cytoplasmic staining, post-neoadjuvant therapy (treated rectal cancer may show decreased or absent MMR protein expression), missense mutation with retained protein antigenicity. Additionally, IHC interpretation may be influenced by poor fixation or technical issues with IHC and is subject to a certain degree of inter-pathologist variability (17). On the other side, molecular assays may be associated with false-positive results due to the presence of variants mimicking unstable alleles or false-negative results caused by the high fragmentation levels of DNA extracted from FFPE samples (18). In this context, ESMO has recently recommended that both MMR-IHC and MSI-PCR testing should be performed in mCRC patients to assess eligibility to treatment with Immune Checkpoint Inhibitors (ICIs) (16).

## Liquid Biopsy in the Management of Colorectal Cancer

**Figure 1:**
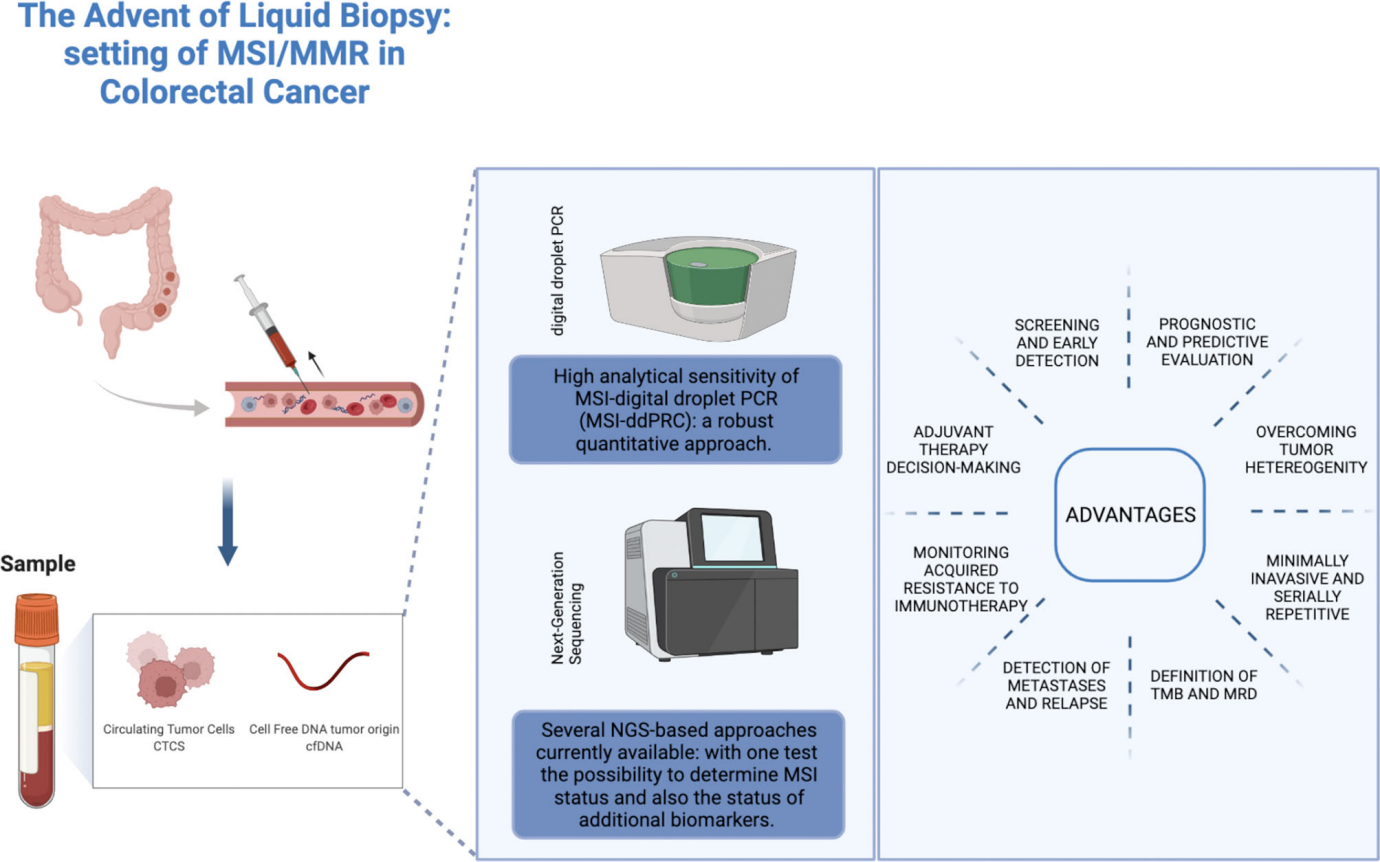
Role of liquid biopsy approaches in the determination of MSI status in colorectal cancer. Credit by Biorender.

The term liquid biopsy is a collective term referred to the sampling and analysis of tumor-derived biomarkers isolated from biological fluids for diagnostic, prognostic, and predictive purposes. The main source for liquid biopsy is peripheral blood; however, urine, cerebrospinal fluid, pleural effusion, and ascites are under investigation as viable alternatives. Liquid biopsy analytes include circulating tumor cells (CTCs), circulating nucleic acids (including circulating tumor DNA [ctDNA], the tumor-derived fraction of cell-free DNA [cfDNA], cell-free RNAs [mRNAs, long non-coding RNAs and microRNAs]), extracellular vesicles, tumor-educated platelets, proteins, and metabolites (19). In this review we will focus on the role of cfDNA and ctDNA in the determination of MSI status (**Figure 1**).

Liquid biopsy has opened unexpected perspectives due to the high concordance with tissue biopsy, the ability to overcome tumor heterogeneity, its non-invasive nature and fast turnaround-time, which renders it a repeatable and reproducible technique, ideal for performing continuous follow-up examinations. However, despite the technological advances, the uptake of liquid biopsy in clinical practice has been slow. At present, several pre-analytical issues limit diagnostic accuracy and sensitivity, with high rates of false positives and negatives. Additionally, the lack of consistency and standardization among different protocols and the absence of multicenter clinical validations and regulatory guidelines hinder the translation of liquid biopsy into clinical practice (20).

Liquid biopsy assays can serve several purposes in the management of CRC. Circulating tumor-derived biomarkers are under investigation for screening and early detection of CRC as alternatives to current secondary prevention methods. In early-stage (stage I-III) and oligometastatic disease, liquid biopsy finds application in prognostic evaluation, adjuvant therapy selection and surveillance for micro-metastatic disease following surgically treatment with curative-intent (i.e., detection of minimal residual disease [MRD]). In metastatic disease, liquid biopsy offers the possibility of identifying predictive biomarkers to guide treatment decision-making through and monitoring response to targeted therapy and immunotherapy (21).

The first important finding in the setting of mCRC is the high concordance rate between molecular profiling performed by ctDNA and tumor tissue analysis (22, 23). Several studies have shown that discordant samples were linked to intra-tumor heterogeneity, previous treatments and/or low tumor burden (24). In this respect, a study presented at ASCO 2022 meeting demonstrated that cfDNA in the mCRC setting can detect biomarkers essential for first-line therapeutic decisions with a frequency comparable to that reported by tissue genotyping. Tissue availability in mCRC does not always guarantee the possibility to perform comprehensive testing quickly, compared to cfDNA. Moreover, oligometastatic disease cannot be adequately evaluated by the molecular point of view by routine biopsy material. As a result, cfDNA has been recently approved by the United States (US) NCCN National Comprehensive Cancer Network as an acceptable alternative to tissue-based genotyping; especially considering that mCRC patients had significantly higher tumor shed compared to other cancer types with guidelines that recommend cfDNA as an option for molecular testing (25, 26).

To date, the principal indications for liquid biopsy in CRC are: i) *RAS* and *BRAF* testing as substitute for tumor tissue analysis in stage IV metastatic CRC and ii) *RAS* mutational profiling for rechallenge in patients resistant to first line anti-EGFR therapies (27).

First-line treatment decision making is a key point in the management of patients with mCRC. At present, according to major guidelines treatment with anti-EGFR therapy is recommended to patients with *RAS* and *BRAF* wild type mCRC (28). Several studies demonstrated the feasibility of performing *RAS* testing on liquid biopsy in daily clinical practice (29). This may be particularly useful when decision on first-line therapy is urgent and tissue recovery from external centers may require a long time (30). Moreover, published data demonstrated that the evaluation of *RAS* mutational status on ctDNA might be helpful in selecting candidate patients with *RAS* wild type mCRC with acquired resistance to anti-EGFR antibodies for a rechallenge strategy (31).

## Liquid Biopsy and the Methods of Detection of MSI

The main technical challenge for liquid biopsy is the low concentration of cfDNA and ctDNA in peripheral blood. Even though CRC is one of the solid tumors shedding the highest amount of ctDNA in the bloodstream, the ratio between ctDNA and cfDNA is variable and the final ctDNA yield can be extremely low, requiring highly sensitive and specific approaches (32).

As a robust quantitative approach, digital-droplet PCR (ddPCR) is being exploited in liquid biopsy testing to detect MSI. This approach is built on a combination of reference probe, that covers target sequences in the flanking region, and drop-off probe that binds microsatellite region. MSI status may be detected by using this approach because mismatch into microsatellite sequence drastically impact on probe hydrolysis. Moreover, ddPCR system enables to separate wild-type from mutated sequences and selectively quantify copies of mutant allele. As regards, a recent paper evaluated analytical performance of ddPCR system in MSI detection rate on three microsatellite makers (*BAT26, ACVR2A*,

*DEFB105A/B*). Results showed that the analytical sensitivity of MSI-ddPCR assays is significantly higher than the detection threshold of the gold standard pentaplex test and a concordance rate of 100% with the reference pentaplex assay for MSI status characterization (33).

Next-Generation Sequencing (NGS) shows great potential for the detection of MSI, allowing the examination of microsatellites at thousands of loci simultaneously, while also obtaining the mutational profile across targeted regions in a single assay. NGS offers the possibility to determine MSI status and also the status of additional biomarkers, such tumor mutational burden (TMB). Moreover, NGS can quantify MSI with reduced noise and reach a sensitivity down to ∼0.05%, compatible with MSI detection in cfDNA and ctDNA (34). As regards, several bioinformatic algorithms based on different computational approaches are currently available. At the first group belong bioinformatics algorithm enabled to detect MSI status by comparing the read-count length distribution of microsatellite sites with matched normal tissue or a reference genome (35). In the second group, computational algorithm based on the implementation of standard data or baseline normal reference were annotated (32). Finally, a third group involves innovative tracer based investigative method for MSI status evaluation. This approach consists in the analysis of mononucleotide repetition longer than 10 bp characterized by a sufficient coverage (default: 20) (36).

There are several NGS-based approaches currently available. Georgiadis and colleagues utilized a hybrid-capture-based 98-kb pan-cancer gene panel that integrates targeted microsatellite regions and TMB analysis. By using a multifactorial error correction method and a novel peak-finding algorithm, it was possible to identify rare MSI frameshift alleles in cfDNA. Results highlighted specificity of >99% and sensitivities of 78% and 67% for MSI and TMB-High, respectively. In addition, clinical outcome also demonstrated that MSI and TMB-High detection could predict progression-free survival (hazard ratios: 0.21 and 0.23, *p*= 0.001 and 0.003, respectively) starting from liquid biopsy analysis of tumor patients (37).

The Guardant360^®^ CDx (Guardant Health, Redwood city, CA, USA) and the FoundationOne^®^ Liquid CDx (Foundation Medicine, Cambridge, MA, USA) are commercial FDA-approved blood-based companion diagnostics that have been adapted for MSI determination in blood samples. The principles of workflow are the same as those of Georgiadis’ method: i) hybrid-capture enrichment of target regions, ii) molecular barcoding to avoid false positives due to technical PCR errors and iii) integration of in silico error correction approaches to reduce background noise. These approaches enable to calculate a MSI score derived from computational analysis that summarizes MSI status in wide mononucleotide genomic loci in comparison with the baseline reference values (21).

Willis and colleagues performed ctDNA testing using the Guardant360^®^ CDx combining 99 putative microsatellite loci and precisely identified 87% of tissue MSI and 99.5% of tissue MSS with an 98.4% overall accuracy and with a limit of detection of 0.1% tumor content. Overall, concordance of cfDNA MSI with tissue PCR and next-generation sequencing was significantly higher than IHC (38).

## Clinical Applications of Liquid Biopsy in the MSI Setting

### Screening and Early Detection

Secondary prevention of CRC cancer is essential to reduce mortality and morbidity rates. Due to its high incidence and large “window of opportunity”, CRC is an optimal candidate for screening. Current screening methods include invasive (i.e., colonoscopy and flexible sigmoidoscopy) and non-invasive methods (fecal occult blood test [FOBT] and fecal immunochemical test [FIT]). Colonoscopy is generally considered the gold standard for the detection of CRC, but it is an invasive technique with higher up-front risks and costs. Although the performance of FOBT and FIT have been substantially improved in recent years, the sensitivity, specificity, and diagnostic accuracy of these methods are still not optimal (39). Since the first report of the presence of ctDNA in the bloodstream of cancer patients in 1989 by Stroun and colleagues, much effort has been made towards the exploitation of ctDNA as a screening technique (40). The main obstacle towards the application of liquid biopsy is the suboptimal limit of detection for small invasive cancers and precancerous lesions (41). Furthermore, the plethora of molecular alterations which characterize CRC challenge the sensitivity of ctDNA as a screening method (42). Since MSI/MMRd is present only in a subset of CRC, is not CRC-specific and is not associated with a specific target population, a MSI-targeted screening approach would be unenforceable and would have no clinical rationale.

At present, gene methylation assays have become promising tool for CRC detection, due to the high concordance between alterations detected in ctDNA and in the primary tumor tissue (43). Additionally, epigenetic alterations, when compared to mutations, are more stable and homogenous within the cancer genome, as they involve specific regions called CpG island. Epigenetic phenomena such as CpG island methylator phenotype (CIMP) are also involved in CRC tumorigenesis, accounting for nearly 10-40% of all sporadic cases; methylation of the promoter of the *MLH1* gene is strongly associated to the CIMP phenotype. Several studies have identified non-invasive methylation biomarkers, which in combination have demonstrated their diagnostic effect in CRC detection, including the following genes: *WIF*, *NPY*, *PENK*, *SEPT9*, *VIM*, *ALX4* (44, 45).

The EpiPRO Colon^®^ is first blood test for cancer screening approved by FDA in 2016 that interrogates methylation status of Septin9 (*SEPT9*) in cfDNA (46). Further investigation reported that the m*SEPT9* test is more sensitive than the FOBT test (47). Some evidence suggests that MMRd status might be associated with methylation of *SEPT9*, thereby this screening test might have a higher specificity for MSI/MMRd CRCs (48). The ColoSure test is a fecal DNA-based test which evaluates vimentin (*VIM*) methylation status with similar diagnostic accuracy (49).

### Detection of MSI as a Predictive and Prognostic Biomarker

MSI and MMRd have emerged as major predictive biomarkers for the efficacy of ICIs in mCRC and are associated with a better survival outcome. As previously stated, tissue-based molecular diagnostics is currently the gold standard for the identification of MSI/MMRd (5).

Unlike for other cancer types, such as non-small-cell lung cancer (NSCLC), tumor tissue availability is not a major issue in the management of mCRC. It is estimated that around 50% of mCRC patients develop metachronous metastases; thus, tissue blocks from the previously resected primary tumor should be available in surgical pathology archives. If the patient presents with synchronous metastases, colonoscopy and/or liver biopsy allow to collect tissue specimen for molecular profiling.

Although tissue specimen is available in the majority of cases, material might be inadequate for immunohistochemical and/or molecular analysis due to small biopsy sample, low tumor content, low tumor cellularity and/or low DNA quality. Additionally, patients often access high volume referral centers at the time of first-line treatment decision making. This situation causes the need of promptly retrieving archival tissue blocks stored elsewhere to collect them at the hub center for molecular testing, in order to select the optimal first-line therapy in advanced stages. For this reason, in the future liquid might represent a valid alternative to perform rapid tumor molecular profiling (30).

Intratumoral heterogeneity is a relevant limitation to the correct biomarker status estimation, especially in the MSI setting. The dynamic process of tumorigenesis occurs through the sequential acquisition of alterations that generate different subpopulations of cells harboring distinct genetic, epigenetic, transcriptomic and/or microenvironment features, which then make up a molecularly heterogeneous tumor. Intratumoral heterogeneity can manifest as spatial heterogeneity at a single disease site (i.e., within the primary tumor or metastatic site) or at distinct disease sites (i.e., between the primary tumor and metastatic site). Intratumoral heterogeneity may fuel resistance to cytotoxic chemotherapy and targeted anticancer agents though the selection of resistant subclones under therapeutic selective pressure (50). A growing body of evidence suggests that genetic and epigenetic intratumor heterogeneity, together with dynamic heterogeneity of the tumor microenvironment has a major impact on the efficacy of various immunotherapies, in particular ICIs (51). A seminal work by Kim and colleagues described MSI/MMR heterogeneity in gastric cancer and its association with a lack of response to pembrolizumab (52). In CRC, MSI/MMR heterogeneity is a relatively rare event and has been described within the primary tumor and between the primary tumor and metastatic site (53–56). A work by Loupakis and colleagues reported the case of a mCRC patient with immunohistochemical and molecular MSI/MMRd heterogeneity in adjacent tumor areas, who achieved deep response to treatment with nivolumab plus ipilimumab (55). While further studies are needed to shed lights on the clinical and therapeutic implications of MSI/MMRd heterogeneity, it is intuitive that tissue specimens (i.e., a single tumor biopsy or a single tissue block of the surgical resection specimen) provide only a limited snapshot of the tumor in time and space, failing to capture tumoral heterogeneity, thus supporting the role of liquid biopsy to identify actionable molecular biomarkers, such as MSI, in mCRC patients.

Clinical trials evaluating feasibility of MSI detection using liquid biopsy are currently ongoing. The NCT03561350 trial is currently evaluating the concordance rate between the electrophoretic mobility profiles of microsatellite biomarkers in cfDNA versus primary tumor tissues in CRC patients exhibiting MSI and testing the hypothesis that changes in the electrophoretic mobility profile of microsatellite biomarkers in liquid biopsies correlate with therapeutic responsiveness to immunotherapy (57). The NCT02563002 trial aims to determine MSI status in the blood sample of advanced CRC patients by using ColonCore NGS panel (58).

Similarly, on the basis of recent molecular classification of gastric adenocarcinoma (GAC) proposed by The Cancer Genome Atlas Research Network, Boldrin et al. compared the analytical performance of different PCR-based approaches (multiplex PCR, real-time PCR and droplet digital PCR) for the detection of MSI status from liquid biopsy specimens previously tested on corresponding tissue specimens. Overall, data confirmed the molecular analysis in liquid biopsy samples as a reliable integrating approach for MSI status evaluation and the digital droplet PCR as the most feasible technique in the analysis of MSI profile from liquid biopsy specimens (59).

However, there are still many limitations challenging the implementation of liquid biopsy in the clinic. The lack of full understanding of the shedding dynamics of tumors hampers the exploitation of liquid biopsy in CRC care. Several factors may cause low ctDNA shedding, including: i) low disease burden, ii) location of metastases (*i.e.*, lung, peritoneum, brain and bone setting are characterized by low shedding), iii) chemotherapy and/or radiotherapy. In these cases, it is crucial to avoid a false negative: an MSS result should be interpreted as such only if adequate tumor related mutations/aberrations were detected in the sample (38, 60).

### Monitoring Acquired Resistance and Response to Immunotherapy

ICIs have led to clinically meaningful improvements in health-related quality of life compared with chemotherapy in MSI CRC patients and are now administered as first-line treatment option in this population. Durable responses suggestive of long-lasting immunologic memory are common among patients treated with ICI. However, a subset of patients showing primary resistance to single-agent ICI therapy can be identified as non-responders. In addition, longer follow-up of clinical trial populations is now revealing late relapses, suggesting the development of acquired resistance (61).

Several studies have demonstrated the predictive value of tracking mutations in liquid biopsy. In this contest, one of the most promising applications of liquid biopsy testing is the possibility to monitor the response to therapy and to track the emergence of resistant subclones withing the tumor cell population (62). Indeed, the loss of ctDNA clones coincides with the loss of MSI in plasma, and the possibility to test MSI non-invasively alongside ctDNA allowed to use both early during one’s treatment to identify responders from non-responders (63).

Liquid biopsy studies have shed light on the mechanisms of acquired resistance to anti-EGFR therapies in mCRCs. *KRAS* mutations are a biomarker for primary resistance to EGFR inhibitors in CRC. In 2012, two seminal studies demonstrated by using liquid biopsy testing that the acquisition of *KRAS* mutations is a mechanism of secondary resistance, thereby limiting clinical benefit and promoting disease progression (64, 65). Subsequent studies demonstrated from the analysis of cfDNA in mCRC patients that acquired resistance to cetuximab are often of polyclonal nature, involving several concomitant genetic alterations (66–68). In 2018, Khan *et al.* demonstrated the first time within a prospective phase II study of anti-EGFR monoclonal antibodies in patients with *RAS* wild type mCRC that the combination of longitudinal plasma profiling can be coupled with mathematical modelling of tumor evolution allows individualized quantitative forecasting of relapse, thus impacting on future clinical decisions (69).

Being minimally invasive, liquid biopsy can be serially repeated in order to ensure real-time analysis of tumor response to immune checkpoint blockade. In a cohort of advanced colorectal or endometrial cancers, MSI allelic frequencies at baseline and at different time points during treatment were found to reflect tumor response to therapy (33). Assessment of the efficacy of response to ICI has proven challenging by using radiographic imaging, particularly due to the phenomenon described as pseudo-progression, which is an initial increase in tumor size, potentially caused by immune cell infiltration, followed by tumor shrinkage. Georgiadis and colleagues found that in a subset of patients with MSI cancers under PD-1 blockade treatment, the residual MSI plasma allele burden was found inversely correlated with the OS and PFS and allowed an earlier prediction of tumor response compared to imaging-based methodologies (37). Particularly of value is the monitoring of response to therapy in patients with MSI tumors. The analysis allows to monitor not only the ctDNA shedding, but also the plasma-MSI status, which levels correlate with response to immunotherapy/ICIs and is cheaper than monitoring ctDNA serially as shown in Kasi PM and colleagues (70, 71). In their works, they reported the clinical utility of identification of MSI in patients with advanced gastrointestinal cancers given the associated approval of multiple ICIs (70, 71). MSI/MMRd is an established biomarker for response to ICIs, but response rates are heterogeneous, and a significant subset of patients do not benefit. Tumor mutational burden is defined as the number of mutations per megabase of DNA (Mut/Mb); high TMB is usually associated with MSI/MMRd and leads to an increase in tumor neoantigens, driving response to immune checkpoint blockade (72, 73). TMB has recently been approved by the FDA as an agnostic biomarker to access treatment with pembrolizumab or dostarlimab (anti-PD-1) (74). Solid tissue analysis is the gold standard for TMB evaluation; however, liquid biopsy approaches, as for MSI, may overcome intratumor heterogeneity (73). In the future, TMB testing on liquid biopsy, alone or in combination with MSI allele burden, may be used to monitor patients’ response to immunotherapy in mCRC. Gandara and colleagues demonstrated that blood-based TMB was able to identify patients who derived clinical benefit in progression-free survival from the anti-PD-L1 atezolizumab in second line and higher NSCLC (75). Georgiardis and colleagues found that not only MSI but also TMB-high in plasma had prognostic value and could predict response to PD-1 blockade (37).

### Figures Minimal Residual Disease (MRD) Detection

Surgery represents the treatment of choice of CRC patients with localized, locally advanced, and also oligometastatic disease. In these patients the detection of ctDNA in plasma samples following surgery can identify the existence of a minimal residual disease, which is a term used to describe a very small number of cancer cells still present after surgery invisible at radio-imaging (76).

In 2008, Diehl and colleagues demonstrated that in CRC patients who had undergone surgical treatment with curative intent, median ctDNA decreased by 97% in less than a day and by 99% within 10 days. On the contrary, if curative resection was not achieved, ctDNA levels decreased much less or increased (77). In the following years, several studies confirmed that the ctDNA could predict recurrence in CRC patients after surgical resections for localized (stage I–III) or oligometastatic disease (78–81). At the recent 2022 ASCO Gastrointestinal Cancer Symposium, the GALAXY study confirmed the ctDNA prognostic role in more than 1500 all stages surgically resected CRC patients (82).

At present, the standard of care for high-risk stage II and stage III CRC patients is adjuvant fluoropyrimidine ± oxaliplatin-based regimens. However, cytotoxic chemotherapy is providing a relatively small net survival advantage of around 3–5% and 10–15% (83). In this setting, ctDNA monitoring appears to be a promising tool under investigation to identify patients with high risk of recurrence after primary tumor resection. In the future, ctDNA might impact post-operative treatment decision-making on top of clinic-pathological factors, such as tumor staging and resection margin status.

It is estimated that about one third of stage III MSI/MMRd CRCs relapse, but with an important prognostic heterogeneity. Improving the prognostication of MSI/MMRd cancer patients is urgently needed to stratify patients according to the risk of recurrence following surgery and to develop specific immunotherapy-based or combinatorial adjuvant therapeutic strategies for this population (83). The “ATOMIC” trial (NCT02912559) is currently evaluating FOLFOX alone or combined with atezolizumab as adjuvant therapy for patients with stage III MMRd or MSI CRC (84).

In this setting, ctDNA might be a useful tool to select patients with early MSI/MMRd CRC at risk of relapse who might benefit from adjuvant immunotherapy alone or in combination, to avoid unnecessary overtreatment. According to the preliminary results of a clinical trial evaluating the use of pembrolizumab following surgery in patients with MSI solid tumors, MRD could be identified in 18% of resected MMRd tumors by ctDNA analysis, suggesting that ctDNA analysis could become a possible tumor agnostic approach for the evaluation of the efficacy of checkpoint blockade in patients at high risk of recurrence (85).

## Conclusions

Liquid biopsy is gaining increasing importance in the management of CRC patients in several clinical settings. However, despite the high number of studies and some promising preliminary results, the use of this approach in clinical practice is still limited. MSI is a well-established prognostic and predictive factor of response to immunotherapy in CRC and is routinely assessed in molecular diagnostics on tissue specimens. In the last decade, advances in sequencing technologies and bioinformatics have dramatically increased the sensitivity of MSI detection in liquid biopsy testing. Although liquid biopsy-based testing to evaluate MSI is still in its early development and has not reached the clinic yet, its potential clinical applications may impact different aspects of the therapeutic decision-making progress in CRC patients. In surgically treatable CRC patients, liquid biopsy MSI testing may help cherry-picking patients who benefit from adjuvant therapy. In the metastatic setting, it is a promising tool to overcome issues associated with tissue-based MMR/MSI assessment (i.e., unavailable, insufficient, or inadequate material) and diagnostic errors caused by intratumoral heterogeneity and to monitor response and resistance onset to immunotherapy.

## Author Contributions

Study concept and design: MF and SL. Acquisition and analysis of data: CC, VA, CR, LP, MS. Interpretation of data: MF, SL, UM, and FB. Drafting of manuscript: CC and VA. Critical revision of the manuscript for important intellectual content: MF, SL, UM, and FB. Manuscript editing: All. Approval to submit: All. All authors have read and agreed to the published version of the manuscript.

## Funding

MF is supported by a grant from the Italian Health Ministry/Veneto Region Research Programme NET-2016–02363853, AIRC 5 per mille 2019 (ID. 22759 programme) and Italian Health Ministry’s Research Program GR-2019-12368903.

## Conflict of Interest

UM has received personal fees (as consultant and/or speaker bureau) from Boehringer Ingelheim, Roche, MSD, Amgen, ThermoFisher Scientifics, Eli Lilly, Diaceutics, GSK, Merck, and AstraZeneca, Jannseen unrelated to the current work. SL has received personal fees for consulting or advisory role from Amgen, Merck Serono, Lilly, Astra Zeneca, Incyte, Daiichi-Sankyo, Bristol-Myers Squibb, Servier, MSD, Roche, Pierre-Fabre, and GSK, and receiving research grants from Amgen, Merck Serono, Bayer, Roche, Lilly, Astra Zeneca, and Bristol-Myers Squibb, unrelated to the current work. Matteo Fassan received research funding from Astellas Pharma, Macrophage Pharma and QED Therapeutics and had roles as consultant or advisor for Astellas Pharma, Roche, Astra Zeneca, MSD and GSK-Tesaro, unrelated to the current work.

The remaining authors declare that the research was conducted in the absence of any commercial or financial relationships that could be construed as a potential conflict of interest.

## Publisher’s Note

All claims expressed in this article are solely those of the authors and do not necessarily represent those of their affiliated organizations, or those of the publisher, the editors and the reviewers. Any product that may be evaluated in this article, or claim that may be made by its manufacturer, is not guaranteed or endorsed by the publisher.
